# Evaluation of Birth by Cesarean Delivery and Development of Early-Onset Colorectal Cancer

**DOI:** 10.1001/jamanetworkopen.2023.10316

**Published:** 2023-04-27

**Authors:** Yin Cao, Long H. Nguyen, Stefani Tica, Ebunoluwa Otegbeye, Xiaoyu Zong, Bjorn Roelstraete, Andrew T. Chan, Barbara B. Warner, Olof Stephansson, Jonas F. Ludvigsson

**Affiliations:** 1 Division of Public Health Sciences, Department of Surgery, Washington University School of Medicine, St Louis, Missouri; 2 Alvin J. Siteman Cancer Center, Washington University School of Medicine, St Louis, Missouri; 3 Division of Gastroenterology, Department of Medicine, Washington University School of Medicine, St Louis, Missouri; 4 Division of Gastroenterology, Massachusetts General Hospital and Harvard Medical School, Boston; 5 Clinical and Translational Epidemiology Unit, Massachusetts General Hospital and Harvard Medical School, Boston; 6 Division of Pediatric Gastroenterology, Hepatology & Nutrition, Department of Pediatrics, Washington University School of Medicine, St Louis, Missouri; 7 Department of Surgery, Washington University School of Medicine, St Louis, Missouri; 8 Department of Medical Epidemiology and Biostatistics, Karolinska Institutet, Stockholm, Sweden; 9 Broad Institute of MIT and Harvard, Cambridge, Massachusetts; 10 Department of Immunology and Infectious Disease, Harvard T.H. Chan School of Public Health, Boston, Massachusetts; 11 Division of Newborn Medicine, Department of Pediatrics, Washington University School of Medicine, St Louis, Missouri; 12 Clinical Epidemiology Division, Department of Medicine, Solna, Karolinska Institutet, Stockholm, Sweden; 13 Division of Women’s Health, Department of Obstetrics, Karolinska University Hospital, Stockholm, Sweden; 14 Department of Paediatrics, Örebro University Hospital, Örebro, Sweden; 15 Department of Medicine, Columbia University Vagelos College of Physicians and Surgeons, New York, New York

## Abstract

**Importance:**

The incidence of early-onset colorectal cancer (CRC), diagnosed younger than 50 years of age, has increased worldwide. Gut dysbiosis throughout the life course is hypothesized as a leading mechanism, yet epidemiologic data are limited.

**Objective:**

To prospectively examine the association between birth by cesarean delivery and early-onset CRC among offspring.

**Design, Setting, and Participants:**

In this population-based, nationwide case-control study in Sweden, adults diagnosed with CRC between 18 and 49 years of age from 1991 to 2017 were identified through the Epidemiology Strengthened by Histopathology Reports in Sweden (ESPRESSO) cohort. Up to 5 general population control individuals without CRC were matched with each case on age, sex, calendar year, and county of residence. Pathology-confirmed end points were linked with the Swedish Medical Birth Register and other national registers. Analyses were conducted from March 2022 through March 2023.

**Exposure:**

Birth by cesarean delivery.

**Main Outcomes and Measures:**

The primary outcome was development of early-onset CRC in the overall population and by sex.

**Results:**

We identified 564 case patients with incident early-onset CRC (mean [SD] age, 32.9 [6.2] years; 284 [50.4%] male) and 2180 matched controls (mean [SD] age, 32.7 [6.3] years; 1104 [50.6%] male). Compared with vaginal delivery, birth by cesarean delivery was not associated with early-onset CRC in the overall population (adjusted odds ratio [aOR], 1.28; 95% CI, 0.91-1.79) after multivariable adjustment for matching and maternal and pregnancy-related factors. A positive association was found for females (aOR, 1.62; 95% CI, 1.01-2.60), but there was no association for males (aOR, 1.05; 95% CI, 0.64-1.72).

**Conclusions and Relevance:**

In this nationwide, population-based case-control study, birth by cesarean delivery was not associated with early-onset CRC compared with birth by vaginal delivery in the overall population in Sweden. However, females born by cesarean delivery had greater odds of early-onset CRC compared with individuals born through vaginal delivery. This finding suggests that early-life gut dysbiosis may contribute to early-onset CRC in females.

## Key Points

**Question:**

Is birth by cesarean delivery associated with risk of early-onset colorectal cancer (CRC)?

**Findings:**

In this nationwide case-control study of 564 individuals with incident early-onset CRC and matched control individuals in Sweden, compared with birth by vaginal delivery, birth by cesarean delivery was not associated with early-onset CRC in the overall population. While no association was observed among males, a positive association was found among females.

**Meaning:**

In this study, females born by cesarean delivery had greater odds of early-onset CRC, suggesting that early-life gut dysbiosis may contribute to early-onset CRC in females.

## Teaser

This case-control study conducted in Sweden examines the association between birth by cesarean delivery and development of early-onset colorectal cancer before age 50 years.

## Introduction

Colorectal cancer (CRC) incidence has increased among individuals younger than 50 years of age in the US^1,2,3^ and multiple European countries, including Sweden,^4^ with more advanced clinicopathological and distinct molecular features compared with CRC in older individuals.^2,5,6,7^ While contributors to the increase in early-onset CRC remain to be identified, obesity,^8,9^ prolonged sitting,^10^ diabetes,^11^ and metabolic syndrome^12^ were all found to be positively associated with early-onset CRC,^5^ supporting in-depth investigations of early-life origins of metabolic dysregulation. The gut microbiome, mechanistically involved in CRC pathogenesis,^13,14,15,16,17,18,19,20,21^ captures substantial variations (approximately 22%-36%) in the aforementioned metabolic traits^22,23,24,25^ and is therefore hypothesized to be at the crossroads between exposome and early-onset CRC.^26,27,28,29^

In addition, in the US, each successive birth cohort has had a higher incidence of early-onset CRC than the previous one.^1^ While the underlying reasons are unknown, this trend supports the hypothesis that early life exposures and changes in exposures throughout the life course over successive birth cohorts may contribute to the rising incidence of early-onset CRC.^30^ Intriguingly, in many developed countries with an increasing incidence of early-onset CRC, a parallel increase in cesarean delivery rates has also been documented.^31^ For instance, in Sweden, the rate of cesarean delivery increased from 5% in 1973 to 12.3% in 1983 and stabilized at 17% in recent years.^32,33^ In the US, the rate of cesarean delivery was 5% between 1950 and the 1970s but rose to 24% in 1986, reached a peak of 33% in 2009, and stabilized around 30% thereafter.^34^ Given the increasing prevalence of birth via cesarean delivery, understanding its associations with future health outcomes has become a critical unmet need. Documented short-term risks associated with birth via cesarean delivery include altered immune development; an increased likelihood of allergy, atopy, and asthma; and reduced intestinal gut microbiome diversity.^35^ Studies on the long-term effects of cesarean delivery in offspring are limited; however, emerging evidence suggests that cesarean delivery may be associated with a higher risk of immune-mediated chronic inflammatory diseases,^36,37^ obesity throughout the life course,^38,39,40,41^ and diabetes,^42^ likely mediated through early-life gut dysbiosis^43,44,45^ that persists throughout adulthood.

Thus far, to our knowledge, the association between birth by cesarean delivery and risk of early-onset CRC has not been examined in epidemiologic studies. To address this critical knowledge gap, we leveraged data collected from Swedish registries to test the hypothesis that birth via cesarean delivery is a factor associated with early-onset CRC.

## Methods

### Study Design and Population

We conducted a nationwide, population-based case-control study using the Epidemiology Strengthened by Histopathology Reports in Sweden (ESPRESSO) cohort. In brief, the ESPRESSO study is a comprehensive data-harmonizing effort involving all 28 pathology departments in Sweden and any gastrointestinal (GI) pathology reports generated for clinical care or research purposes between 1965 and 2017.^46^ This consortium has enrolled more than 2.1 million unique individuals with detailed information on GI topography, morphologic appearance, and pathologist’s diagnostic impression. The unique Swedish personal identity number was used to link ESPRESSO data to several national registers containing validated, prospectively recorded data on demographics and disease diagnoses^47^ and the Swedish Medical Birth Register (MBR), with data on mode of birth since 1973. The MBR covers around 98% of all births in Sweden and contains data from the first antenatal visit until delivery and discharge from the delivery hospital. Completeness of pregnancy and neonatal outcomes in the MBR is bolstered by cross-comparison with the Total Population Register to identify missing records and standardization of electronic health records across the country as well as by Sweden’s universally accessible obstetric care.^48,49^ This study was approved by the Stockholm Ethics Review Board. Informed consent was waived, as the study was registry based. This study followed the Strengthening the Reporting of Observational Studies in Epidemiology (STROBE) reporting guideline for case-control studies.

### Ascertainment of Incident Early-Onset CRC Cases and Matched Controls

We identified individuals in the ESPRESSO study with GI tract histopathologic findings compatible with a diagnosis of incident CRC between age 18 and 49 years from 1991 to 2017 (eTable 1 in Supplement 1). We then cross-referenced potential cases with their inpatient and outpatient records for *International Classification of Diseases, Seventh Revision (ICD-7)*, *Eighth Revision (ICD-8)*, and *Ninth Revision (ICD-9)* and *International Statistical Classification of Diseases and Related Health Problems, Tenth Revision (ICD-10)* codes consistent with CRC (eTable 1 in Supplement 1), thus requiring both compatible histopathologic findings and registry-level case confirmation. Information on the date of diagnosis and tumor location were retrieved. Incident cases of CRC were matched with up to 5 control individuals from the general population based on age at index (continuous), sex (categorical), calendar year (continuous), and county of residence^50^ (categorical).^46^ We excluded cases and controls with prior inflammatory bowel disease (IBD) and hereditary cancers.

### Assessment of Mode of Delivery and Maternal Factors

We identified mothers to cases and controls through the MBR and extracted information on the mode of birth. For covariates, a list of maternal and pregnancy-related factors was selected a priori, including maternal history of cesarean delivery, maternal age at delivery, maternal country of birth, whether the mother lived with a partner, maternal educational level, and parity. Birth characteristics were also extracted from the MBR, including gestational age (weeks), birth weight (grams), and birth length (centimeters). We leveraged both the MBR and the National Patient Register to retrieve information on diagnoses of maternal comorbidities, including diabetes (pregestational and gestational), hypertension, preeclampsia, and IBD.

### Statistical Analysis

We evaluated the association between birth by cesarean delivery and odds of early-onset CRC as the primary analysis. As secondary analyses, we investigated whether the findings in the primary analysis differed according to sex and anatomic site of the tumor.

Multivariable conditional logistic regression was used to estimate adjusted odds ratios (aORs) and 95% CIs. In addition to condition on matching factors (age, sex, calendar year, and county of residence), we adjusted for maternal and pregnancy-related factors as the main multivariable analyses: previous cesarean delivery (yes, no), maternal age (≤24, 25-29, 30-34, or ≥35 years), maternal country of birth (Nordic, non-Nordic), living with a partner (yes, no), maternal educational level (elementary, secondary, or college), and parity (1, 2, 3, or ≥4). Additionally, in a separate model, we adjusted for birth characteristics, including gestational age (<36, 37-39, 40-42, or ≥43 weeks), birth weight^51^ (<2500, 2500 to <3000, 3000 to <3500, 3500 to <4000, or ≥4000 g), and birth length^52^ (continuous), to assess whether the findings were mediated by these factors. Missingness was minimal (only for maternal educational level, gestational age, birth weight, and length, all less than 5%), and missing data were imputed using multiple imputation by chained equations methods with 5 iterations.^53^

We conducted the following sensitivity analyses: (1) restricted to cases and controls aged 35 years or older to further minimize the impact of hereditary CRCs, (2) restricted to cases and controls without a maternal history of cesarean delivery to minimize potential confounding, and (3) restricted to cases and controls without maternal history of diabetes, gestational diabetes, hypertension, preeclampsia, and IBD up to the time of delivery to minimize confounding. E-values, which quantify the minimum effect required for an unmeasured confounder on both exposure and outcome to nullify the observed exposure-outcome associations, were calculated to evaluate the effects of unmeasured confounding.^54,55^ All analyses were performed in SAS, version 9.4 (SAS Institute Inc). Data were analyzed from March 2022 through March 2023.

## Results

We identified 705 individuals aged 18 to 49 years with incident early-onset CRC in ESPRESSO from 1991 to 2017 and 3509 eligible controls after matching on age, sex, calendar year, and county of residence. After linking with birth records, a total of 564 cases (mean [SD] age, 32.9 [6.2] years; 280 [49.6%] female and 284 [50.4%] male; 154 [27.3%] with rectal cancer) and 2180 controls (mean [SD] age, 32.7 [6.3] years; 1076 [49.4%] female and 1104 [50.6%] male) remained in the final analyses (Table 1, Table 2, and eFigure in Supplement 1), with similar characteristics as those among cases and controls not linked with birth records (eTable 2 in Supplement 1). All of the included 564 cases were matched with at least 1 control, with the majority (505 [89.5%]) matched with 3 to 5 controls. Compared with mothers of matched controls, mothers of the case patients had similar age at delivery and distribution of country of birth, proportion who lived with a partner, educational level, and parity but were more likely to have a history of cesarean delivery, diabetes, and IBD. No apparent differences were observed for gestational length, birth weight, and birth length. A higher percentage of early-onset CRC cases (55 of 564 [9.8%]) were born through cesarean delivery compared with controls (175 of 2180 [8.0%]).

**Table 1.** Characteristics of Patients With Incident Early-Onset Colorectal Cancer and Matched Control Individuals in Sweden From 1991 to 2017

Characteristic	Individuals^a^	
	Cases (n = 564)	Controls (n = 2180)
**Matching factors**		
Age at index date, mean (SD), y	32.9 (6.2)	32.7 (6.3)
Sex		
Female	280 (49.6)	1076 (49.4)
Male	284 (50.4)	1104 (50.6)
Year of birth		
1973-1979	413 (73.2)	1606 (73.7)
1980-1989	133 (23.6)	503 (23.1)
1990-1997	18 (3.2)	71 (3.3)
Year at index date		
1993-2002	39 (6.9)	176 (8.1)
2003-2012	266 (47.2)	1052 (48.3)
2013-2017	259 (45.9)	952 (43.7)
Mode of birth		
Cesarean delivery	55 (9.8)	175 (8.0)
Vaginal delivery	509 (90.2)	2005 (92.0)
**Maternal and pregnancy-related factors**		
Maternal age at delivery, y		
Mean (SD)	26.4 (4.8)	26.7 (4.9)
≤24	213 (37.8)	752 (34.5)
25-29	210 (37.2)	829 (38.0)
30-34	102 (18.1)	457 (21.0)
≥35	39 (6.9)	142 (6.5)
Parity, mean (SD), No.	1.8 (1.0)	1.8 (1.0)
Living with a partner	320 (56.7)	1214 (55.7)
Maternal educational level		
Elementary	139 (24.6)	567 (26.0)
Secondary	294 (52.1)	1099 (50.4)
College	119 (21.1)	486 (22.3)
Missing	12 (2.1)	28 (1.3)
Maternal country of birth		
Nordic	539 (95.6)	2107 (96.7)
Non-Nordic	25 (4.4)	73 (3.3)
Maternal diabetes	4 (0.7)	9 (0.4)
Gestational diabetes	1 (0.2)	3 (0.1)
Pregestational hypertension	1 (0.2)	10 (0.5)
Preeclampsia	1 (0.2)	5 (0.2)
Maternal inflammatory bowel disease	2 (0.4)	3 (0.1)
Maternal history of cesarean delivery	17 (3.0)	49 (2.2)
**Birth characteristics**		
Gestational age, wk		
Mean (SD)	39.7 (1.8)	39.6 (1.9)
≤36	19 (3.4)	108 (5.0)
37-39	210 (37.2)	792 (36.3)
40-42	319 (56.6)	1214 (55.7)
≥43	13 (2.3)	55 (2.5)
Missing	3 (0.5)	11 (0.5)
Birth weight, g		
Mean (SD)	3490 (556)	3480 (533)
<2500	22 (3.9)	86 (3.9)
2500-2999	69 (12.2)	244 (11.2)
3000-3499	191 (33.9)	749 (34.4)
3500-3999	185 (32.8)	757 (34.7)
≥4000	94 (16.7)	342 (15.7)
Missing	3 (0.5)	2 (0.1)
Birth length, mean (SD), cm	50.4 (2.4)	50.4 (2.4)

^a^ Data are presented as the number (percentage) of individuals unless otherwise indicated.

**Table 2.** Characteristics of Patients With Incident Early-Onset Colorectal Cancer in Sweden From 1991 to 2017

Characteristic	Patients (N = 564)^a^
Age at index date, y	
Mean (SD)	33 (6.2)
18-29	193 (34.2)
30-39	307 (54.4)
≥40	64 (11.3)
Anatomic site	
Proximal colon	44 (7.8)
Distal colon	68 (12.1)
Colon unspecified	298 (52.8)
Rectal	154 (27.3)

^a^ Data are presented as the number (percentage) of patients unless otherwise indicated.

Overall, individuals who were born through cesarean delivery did not have significantly higher odds of early-onset CRC (aOR, 1.28; 95% CI, 0.91-1.79) (Table 3) after multivariable adjustment of matching and maternal and pregnancy factors, including previous cesarean delivery, maternal age, country of birth, living with a partner, educational level, and parity. A positive association was found among females (aOR, 1.62; 95% CI, 1.01-2.60), but there was no association among males (aOR, 1.05; 95% CI, 0.64-1.72). An unmeasured confounder would need to be associated with both birth via cesarean delivery and early-onset CRC with an OR of at least 2.62 to nullify the association observed among females (eTable 3 in Supplement 1). We also observed similar associations after further adjusting for birth characteristics, including gestational age, birth weight, and birth length (Table 3).

**Table 3.** Birth via Cesarean Delivery and Risk of Early-Onset Colorectal Cancer

Group	Vaginal delivery	Cesarean delivery
**All participants**		
Cases:controls, No.	509:2005	55:175
Model 1: OR (95% CI)^a^	1 [Reference]	1.25 (0.90-1.73)
Model 2: aOR (95% CI)^b^	1 [Reference]	1.28 (0.91-1.79)
Model 3: aOR (95% CI)^c^	1 [Reference]	1.32 (0.93-1.86)
**Females**		
Cases:controls, No.	249:991	31:85
Model 1: OR (95% CI)^a^	1 [Reference]	1.50 (0.96-2.33)
Model 2: aOR (95% CI)^b^	1 [Reference]	1.62 (1.01-2.60)
Model 3: aOR (95% CI)^c^	1 [Reference]	1.64 (1.01-2.69)
**Males**		
Cases:Controls, No.	260:1014	24:90
Model 1: OR (95% CI)^a^	1 [Reference]	1.03 (0.64-1.66)
Model 2: aOR (95% CI)^b^	1 [Reference]	1.05 (0.64-1.72)
Model 3: aOR (95% CI)^c^	1 [Reference]	1.09 (0.66-1.81)

Abbreviation: aOR, adjusted odds ratio.

^a^ Model 1 was conditioned on matching factors, including age at index date (continuous), sex, calendar year of index date (continuous), and county of residence.

^b^ Model 2 was additionally adjusted for maternal and pregnancy-related factors at delivery: maternal history of cesarean delivery (yes, no), maternal age at delivery (≤24, 25-29, 30-34, or ≥35 years), maternal country of birth (Nordic, non-Nordic), living with a partner (yes, no), maternal educational level (elementary, secondary, or college), and parity (1, 2, 3, or ≥4).

^c^ Model 3 was additionally adjusted for birth characteristics, including gestational age (<36, 37-39, 40-42, or ≥43 weeks), birth weight (<2500, 2500 to <3000, 3000 to <3500, 3500 to <4000, and ≥4000 g), and birth length (continuous).

For females, the association was also similar after restricting to those aged 35 years or older at the index date (aOR, 2.13; 95% CI, 1.02-4.48), those without maternal history of cesarean delivery (aOR, 1.63; 95% CI, 1.00-2.66), or those without maternal history of diabetes, gestational diabetes, hypertension, preeclampsia, or IBD (aOR, 1.70; 95% CI, 1.05-2.74) (Table 4).

**Table 4.** Sensitivity Analyses of Birth via Cesarean Delivery and Risk of Early-Onset Colorectal Cancer

Analysis	Vaginal delivery	Cesarean delivery
**Age at index ≥35 y**		
All		
Cases:controls, No.	234:872	21:70
aOR (95% CI)^a^	1 [Reference]	1.19 (0.70-2.05)
Females		
Cases:controls, No.	109:407	13:29
aOR (95% CI)^a^	1 [Reference]	2.13 (1.02-4.48)
Males		
Cases:controls, No.	125:465	8:41
aOR (95% CI)^a^	1 [Reference]	0.71 (0.30-1.67)
**Without maternal history of cesarean delivery**		
All		
Cases:controls, No.	503:1978	44:153
aOR (95% CI)^a^	1 [Reference]	1.22 (0.85-1.74)
Females		
Cases:controls, No.	246:983	26:73
aOR (95% CI)^a^	1 [Reference]	1.63 (1.00-2.66)
Males		
Cases:controls, No.	257:995	18:80
aOR (95% CI)^a^	1 [Reference]	0.89 (0.52-1.53)
**Without maternal history of major comorbidities^b^**		
All		
Cases:controls, No.	504:1988	51:163
aOR (95% CI)^a^	1 [Reference]	1.30 (0.92-1.83)
Females		
Cases:controls, No.	246:982	30:79
aOR (95% CI)^a^	1 [Reference]	1.70 (1.05-2.74)
Males		
Cases:controls, No.	258:1006	21:84
aOR (95% CI)^a^	1 [Reference]	1.00 (0.59-1.69)

Abbreviation: aOR, adjusted odds ratio.

^a^ The model was conditioned on matching factors, including age at index date (continuous), sex, calendar year of index date (continuous), and county of residence, and additionally adjusted for maternal and pregnancy-related factors: maternal history of cesarean delivery (yes, no), maternal age at delivery (≤24, 25-29, 30-34, or ≥35 years), maternal country of birth (Nordic, non-Nordic), living with partner (yes, no), maternal educational level (elementary, secondary, or college), and parity (1, 2, 3, or ≥4).

^b^ Maternal history of major comorbidities included diabetes, gestational diabetes, hypertension, preeclampsia, and inflammatory bowel disease.

We further examined whether the positive association for females differed according to anatomic sites of CRC (colon vs rectal). For colon cancer, the aOR was 1.77 (95% CI, 1.05-3.01), while for rectal cancer, the aOR was 1.29 (95% CI, 0.42-4.02) (eTable 4 in Supplement 1).

## Discussion

In this population-based case-control study that included 564 patients with early-onset CRC, we found no association between birth by cesarean delivery and early-onset CRC compared with birth by vaginal delivery in the overall population. However, we identified an association between birth by cesarean delivery and higher odds of early-onset CRC among females, and this association was not mediated by birth characteristics. To our knowledge, this is among the first analyses leveraging prospectively collected data to examine the association between birth by cesarean delivery and risk of CRC, with a focus on early-onset CRC. Although preliminary, our findings lend initial support to the hypothesis that early-life gut dysbiosis may contribute to the rising incidence of early-onset CRC in females, calling for validation and mechanistic studies. If validated, modeling studies^56^ are needed to elucidate the contribution of cesarean delivery to the rising incidence of early-onset CRC, especially among women.

The mechanisms linking birth by cesarean delivery to higher risk of early-onset CRC remain unexplored, although effects on the developing gut microbiome are a suspected mediator.^26^ Among various perinatal factors affecting the transfer of the maternal microbiome, cesarean delivery exerts the strongest disruption, affecting both the diversity and the composition of early infancy microbiome,^57^ with reported differences persisting through the first year of life.^43,45,58,59^ For instance, gut bacterial colony load was significantly less at birth in infants born by cesarean delivery, with relatively reduced abundance of *Bacteroides* and *Bifidobacterium* species up to 6 months of life.^43,60^ While preclinical studies have shown *Bifidobacterium* species to have anticancer properties in CRC through different mechanisms,^61,62^ longitudinal studies with long-term follow-up and repeated microbiome profiling are needed to uncover the evolutionary dynamics of affected species and strains,^63^ including interaction with host genetics.^64^ Furthermore, early infancy is considered a critical period during which microbial differences significantly influence immune maturation.^43,65,66,67^ In particular, the colon is the site of the greatest microbial colonization by cell count and biomass and possesses the largest immune exposure by density and surface area.^68,69^ Accumulating evidence suggests that deficient or aberrant immune maturation may have an association with disease development later in life,^70,71,72^ such as with the development of IBD—a condition that also has been associated with early-onset CRC.^73^

Cesarean delivery has been associated with modest increased risk of obesity^38,39,40,41,42,74^ and diabetes,^37,42^ both of which are factors associated with early-onset CRC.^5,8,9,12^ Recent evidence suggested that adolescents born by cesarean delivery had significantly lower levels of adiponectin and higher levels of insulin resistance compared with adolescents born by vaginal delivery,^75^ further supporting the possibility that cesarean delivery may be associated with higher subsequent risk of early-onset CRC. More research is needed to elucidate the complex interplays between cesarean delivery, insulin resistance, and the microbiome-immune-cancer axis throughout the life course.

Notably, we observed an association between birth by cesarean delivery and early-onset CRC among females but not among males. Although the rise in early-onset CRC in Sweden was similar in women and men,^76,77^ our findings suggest that risk factors and/or strengths of associations may differ by sex. Sex dimorphism has previously been observed in associations between cesarean delivery and chronic diseases. A prior study from Kaiser Permanente’s Northwest Region (US) reported an elevated risk of asthma among females (aOR, 1.53; 95% CI, 1.11-2.10) but not among males (aOR, 1.08; 95% CI, 0.81-1.43) born via cesarean delivery.^78^ While evidence in humans is thus far limited, preclinical work suggests that sex hormones influence interactions between microbial signaling, the enteric immune system, and mucosal barrier functioning.^79^ However, whether this physiology influences the development of early-onset CRC represents an important area of future study. To date, there is also a paucity of data exploring multigenerational trends in the microbiome and influence on disease development, although some researchers have hypothesized this may play a role in mother-daughter dyads and disease trends as rates of cesarean delivery have increased.^80,81^

### Strengths and Limitations

Strengths of our study include the use of national registries with extended follow-up to prospectively examine the association between birth via cesarean delivery and the odds of early-onset CRC, such that the findings are not influenced by maternal recall bias. Further, accuracy of CRC diagnosis may have been increased by the requirement of congruency in histopathologic findings and diagnostic codes. We were also able to adjust for a list of maternal factors to account for potential confounding.

There are also several limitations to this study. First, even though we leveraged a nationwide population-based cohort, the rate of cesarean delivery was relatively low in Sweden between 1970 and the 1980s compared with many developed countries, limiting our sample size and power, especially for additional analyses by CRC anatomic sites and elective vs emergency cesarean delivery (information available since 1999). As the mother usually does not experience rupture of the amniotic membrane until surgery, a newborn delivered by elective cesarean delivery has limited microbial colonization from the birth canal compared with a newborn born via emergency cesarean delivery, which is usually performed after the onset of physical labor and the rupture of membranes.^82^ A prior study suggested that elective cesarean delivery but not emergency cesarean delivery was associated with an increased risk of childhood acute lymphoblastic leukemia.^83^ Future studies are needed to elucidate whether this pertains to CRC and/or early-onset CRC. In addition, we did not adjust for indications for cesarean delivery (eg, fetopelvic disproportion, breech presentation, or fetal distress^32^) unless they were known to be associated with risk of CRC, such as IBD. Second, no information on antibiotic prophylaxis for cesarean delivery and intrapartum antibiotic use was available. Although concerns about early-life exposure to broad-spectrum antibiotics and associated pervasive effects on the development of the gut microbiome and various disorders later in life are growing,^84,85^ long-term data are limited. A recent randomized clinical trial^86^ compared the microbiome composition of infants born via cesarean delivery with and without intrauterine antibiotic exposure and reported that cesarean delivery itself, but not antenatal antibiotic exposure, negatively affected microbiota development. Finally, residual confounding (eg, socioeconomic status of the family) could not be ruled out. Our findings were generally robust, supported by E-value analyses and sensitivity analyses including restricting to individuals without maternal history of a list of major comorbidities. We also attempted to conduct sibling analyses; however, only 6% of sibling pairs had different modes of delivery, and this study was underpowered to evaluate the association among siblings. Lack of information on maternal adiposity and weight gain during pregnancy^87^ (body mass index data were only available since 1992) limited our capability to further assess their roles in the association identified. Validations in more racially and ethnically diverse populations, especially from other countries with rising incidence of early-onset CRC, are needed.

## Conclusions

In this population-based case-control study, compared with birth by vaginal delivery, birth by cesarean delivery was not associated with early-onset CRC in the overall population in Sweden. Females born via cesarean delivery had greater odds of developing early-onset CRC compared with individuals born through vaginal delivery, but there was no association among males.

## Notes

Supplement 1. **eTable 1.** Histopathology and *International Classification of Diseases *(*ICD*) to Identify Colorectal Cancer **eTable 2.** Characteristics of Excluded Cases and Controls Due to Lack of Birth Records, Sweden, 1991-2017 **eTable 3.** E-Values for the Association Between Birth via Cesarean Delivery and Risk of Early-Onset Colorectal Cancer **eTable 4.** Birth via Cesarean Delivery and Risk of Early-Onset Colorectal Cancer According to Anatomic Site **eFigure.** Flow Chart of Study Population

Supplement 2.
Data Sharing Statement
