# Supplementary material for: Evaluation of Birth by Cesarean Delivery and Development of Early-Onset Colorectal Cancer
Source: JAMA Netw Open. 2023 Apr 27;6(4):e2310316. doi: 10.1001/jamanetworkopen.2023.10316 (PMC10140807; doi:10.1001/jamanetworkopen.2023.10316)
Supplement: Supplement 1. — eTable 1. Histopathology and International Classification of Diseases (ICD) to Identify Colorectal Cancer eTable 2. Characteristics of Excluded Cases and Controls Due to Lack of Birth Records, Sweden, 1991-2017 eTable 3. E-Values for the Association Between Birth via Cesarean Delivery and Risk of Early-Onset Colorectal Cancer eTable 4. Birth via Cesarean Delivery and Risk of Early-Onset Colorectal Cancer According to Anatomic Site eFigure. Flow Chart of Study Population [file jamanetwopen-e2310316-s001.pdf]

Supplementary Online Content

Cao Y, Nguyen LH, Tica S, et al. Evaluation of birth by cesarean delivery and development of early-onset colorectal cancer. *JAMA Netw Open*. 2023;6(4):e2310316. doi:10.1001/jamanetworkopen.2023.10316

eTable 1. Histopathology and *International Classification of Diseases* (*ICD*) to Identify Colorectal Cancer
eTable 2. Characteristics of Excluded Cases and Controls Due to Lack of Birth Records, Sweden, 1991-2017
eTable 3. E-Values for the Association Between Birth via Cesarean Delivery and Risk of Early-Onset Colorectal Cancer
eTable 4. Birth via Cesarean Delivery and Risk of Early-Onset Colorectal Cancer According to Anatomic Site
eFigure. Flow Chart of Study Population

This supplementary material has been provided by the authors to give readers additional information about their work.

© 2023 Cao Y et al. *JAMA Network Open*.

eTable 1. Histopathology and International Classification of Diseases (ICD) to identify colorectal cancer

		Colorectal cancer (CRC)
Histopathology	Anatomic location	T67X (colon), T671-674 (proximal colon), T675-677 (distal colon), T68X (rectum)
	Histologic features (morphology codes)	M81400, M81401, M81402, M81403, M814031, M814032, M814033, M81404, M81406, M814062, M81407, M81409, M814093, M81443, M81453
Diagnostic codes from the Swedish Patient Register	ICD-7/8/9	153.X, 154.X
	ICD-10	C18, C19, C20

Abbreviation: ICD, international classification of disease

© 2023 Cao Y et al. *JAMA Network Open*.

eTable 2. Characteristics of excluded cases and controls due to lack of birth records, Sweden (1991-2017).

Characteristics	Cases, No. (%)	Controls without birth records, No. (%)	Controls matched to cases without birth records, No. (%)
Total No.	141	803	526
Age at index date, y			
Mean (SD)	32.8 (5.6)	33.8 (5.6)	32.7 (5.8)
Sex			
Female	63 (44.7)	404 (50.3)	225 (42.8)
Male	78 (55.3)	399 (49.7)	301 (57.2)
Year of birth			
1973-1979	102 (72.3)	577 (71.9)	385 (73.2)
1980-1989	34 (24.1)	209 (26.0)	120 (22.8)
1990-1997	5 (3.55)	17 (2.12)	21 (3.99)
Year at index date			
1993-2002	5 (3.6)	21 (2.6)	22 (4.2)
2003-2012	70 (49.6)	357 (44.5)	260 (49.4)
2013-2017	66 (46.8)	425 (52.9)	244 (46.4)
Country of birth			
Sweden	4 (2.8)	24 (3.0)	526 (100)
Nordic except Sweden	3 (2.1)	37 (4.6)	-
Non-Nordic	134 (95.0)	742 (92.4)	-

Abbreviation: SD, standard deviation.

© 2023 Cao Y et al. *JAMA Network Open*.

eTable 3. E-values for the association between birth via cesarean delivery and risk of early-onset colorectal cancer.

	aOR (95% CI) [a]	E-Value (Point Estimate)	E-Value (Confidence Interval)[b]
**All participants**	1.28 (0.91-1.79)	1.88	1.00
**Females**	1.62 (1.01-2.60)	2.62	1.11
**Males**	1.05 (0.64-1.72)	1.28	1.00

[a] aORs were from model 2 in Table 3. The E-value quantifies the minimum strength of association required between an unmeasured confounder and both exposure and outcome, with consideration of measured covariates, to nullify the observed exposure-outcome associations. E-values were calculated using a publicly available and validated online calculator.

[b] E-values for the confidence interval limit closest to the null.

Abbreviation: aOR, adjusted odds ratio; CI, confidence interval.

© 2023 Cao Y et al. *JAMA Network Open*.

eTable 4. Birth via cesarean delivery and risk of early-onset colorectal cancer according to anatomic site.

	Vaginal delivery	Cesarean delivery
**Females**		
**Colon cancer**		
Cases:Controls, No.	186:727	26:65
aOR (95% CI)[a]	1 [Reference]	1.77 (1.05-3.01)
**Rectal cancer**		
Cases:Controls, No.	63:264	5:20
aOR (95% CI)[a]	1 [Reference]	1.29 (0.42-4.02)
**Males**		
**Colon cancer**		
Cases:Controls, No.	181:706	17:64
aOR (95% CI)[a]	1 [Reference]	1.04 (0.58-1.85)
**Rectal cancer**		
Cases:Controls, No.	79:308	7:26
aOR (95% CI)[a]	1 [Reference]	1.01 (0.38-2.67)

[a] The model was conditioned on matching factors, including age at index date (continuous), sex, calendar year of index date (continuous), and county of residence, and additionally adjusted for maternal and pregnancy-related factors: maternal history of cesarean delivery (yes, no), maternal age at delivery (≤24, 25-29, 30-34, ≥35), maternal country of birth (Nordic, non-Nordic), living with partner (yes, no), maternal educational level (elementary, secondary, college), and parity (1,2,3, ≥4).

Abbreviation: aOR: adjusted odds ratio; CI: confidence interval.

© 2023 Cao Y et al. *JAMA Network Open*.

eFigure. Flow chart of study population.

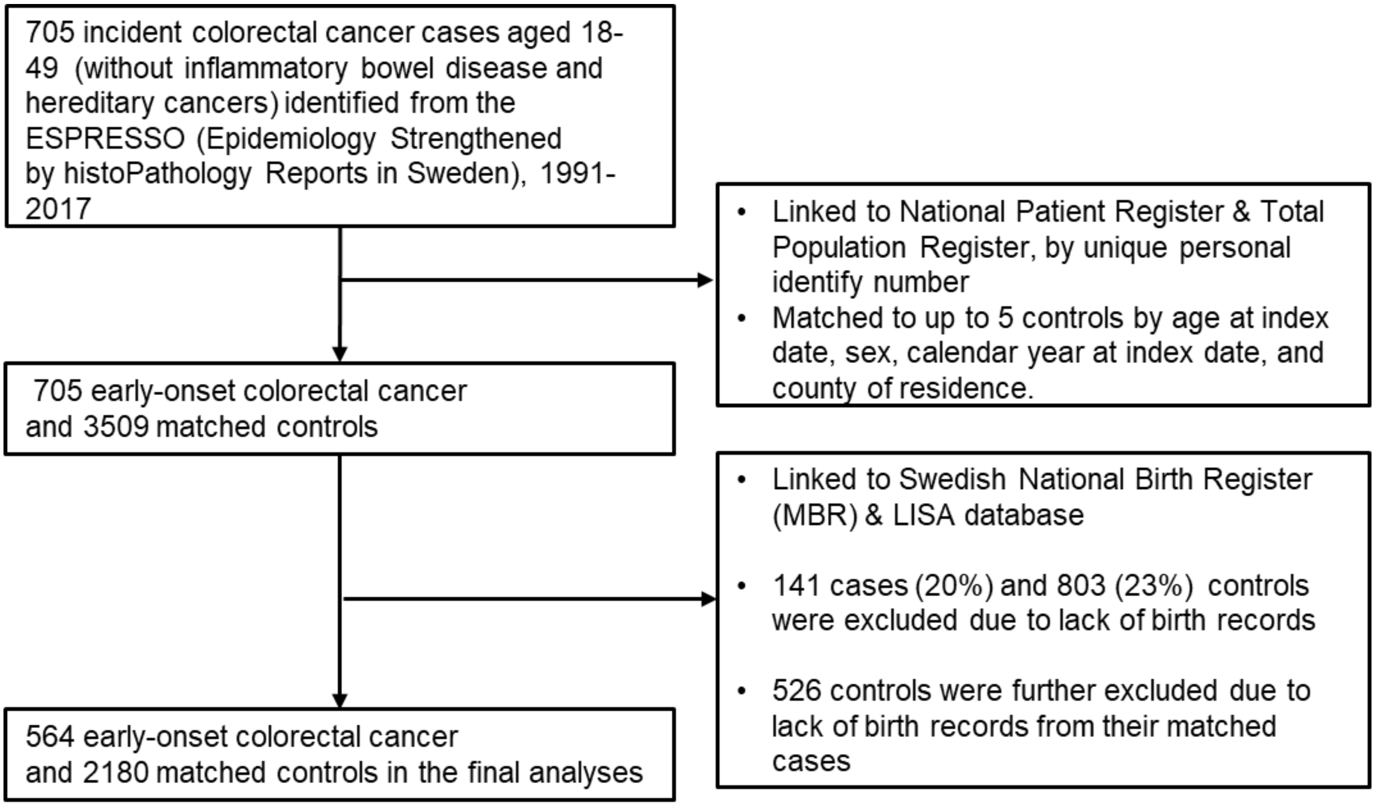

© 2023 Cao Y et al. *JAMA Network Open*.
