# Supplementary material for: Evaluation of Birth by Cesarean Delivery and Development of Early-Onset Colorectal Cancer
Source: JAMA Netw Open. 2023 Apr 27;6(4):e2310316. doi: 10.1001/jamanetworkopen.2023.10316 (PMC10140807; doi:10.1001/jamanetworkopen.2023.10316)
Supplement: Supplement 2. — Data Sharing Statement [file jamanetwopen-e2310316-s002.pdf]

Data Sharing Statement

Cao. Evaluation of Birth by Cesarean Delivery and Development of Early-Onset Colorectal Cancer. *JAMA Netw Open*. Published April 27, 2023.
doi:10.1001/jamanetworkopen.2023.10316

Data
Data available: No
